# Low and moderate-fat plant sterol fortified soymilk in modulation of plasma lipids and cholesterol kinetics in subjects with normal to high cholesterol concentrations: report on two randomized crossover studies

**DOI:** 10.1186/1476-511X-8-45

**Published:** 2009-10-20

**Authors:** Todd C Rideout, Yen-Ming Chan, Scott V Harding, Peter JH Jones

**Affiliations:** 1 Richardson Centre for Functional Foods and Nutraceuticals, University of Manitoba, Winnipeg, Manitoba R3T 6C5, Canada

## Abstract

**Background:**

Although consumption of various plant sterol (PS)-enriched beverages is effective in lowering plasma cholesterol, the lipid-lowering potential of PS in a soymilk format has not been investigated thoroughly. Therefore, to evaluate the efficacy of PS-enriched soy beverages on plasma lipids and cholesterol kinetics, we conducted two separate 28 d dietary controlled cross-over studies. In study 1, the cholesterol-lowering efficacy of a low-fat (2 g/serving) PS enriched soy beverage was examined in 33 normal cholesterolemic subjects in comparison with 1% dairy milk. In study 2, we investigated the efficacy of a moderate-fat (3.5 g/serving) PS-enriched soy beverage on plasma cholesterol concentrations and cholesterol kinetic responses in 23 hypercholesterolemic subjects compared with 1% dairy milk. Both the low and moderate-fat PS-enriched soymilk varieties provided 1.95 g PS/d. Endpoint plasma variables were analyzed by repeated-measures ANOVA using baseline values as covariates for plasma lipid measurements.

**Results:**

In comparison with the 1% dairy milk control, the low-fat soy beverage reduced (*P *< 0.05) total and LDL-cholesterol by 10 and 13%, respectively. Consumption of the moderate-fat PS-enriched soy beverage reduced (*P *< 0.05) plasma total and LDL-cholesterol by 12 and 15% respectively. Fasting triglycerides were reduced by 9.4% following consumption of the moderate-fat soy beverage in comparison with the 1% dairy milk. Both low and moderate-fat PS-enriched soy varieties reduced (*P *< 0.05) LDL:HDL and TC:HDL ratios compared with the 1% dairy milk control. Consumption of the moderate-fat PS-enriched soymilk reduced (*P *< 0.05) cholesterol absorption by 27%, but did not alter cholesterol synthesis in comparison with 1% dairy milk.

**Conclusion:**

We conclude that, compared to 1% dairy milk, consumption of low and moderate-fat PS-enriched soy beverages represents an effective dietary strategy to reduce circulating lipid concentrations in normal to hypercholesterolemic individuals by reducing intestinal cholesterol absorption.

**Trial registration (clinicaltrials.gov):**

NCT00923403 (Study 1), NCT00924391 (Study 2).

## Background

Although drug treatment is effective in the management of dyslipidemia, diet-based monotherapies or combination dietary approaches that reduce the incidence of arterial vascular diseases will be integral to sustainable health care systems of the future [1]. Consumption of plant sterols (PS), constituents found in plants that chemically resemble cholesterol, is a well-defined dietary lipid-lowering strategy with repeated demonstrations of cholesterol reductions in human clinical investigations in the range of 8-15% [2-4]. However, it is increasingly clear that the food/beverage format used as the PS delivery vehicle may impact PS solubility and hence modulate intestinal cholesterol absorption and the magnitude of cholesterol-lowering [5,6]. For instance, although cholesterol reductions are observed following consumption of PS-fortified products including juice [7], margarine [8], and dressings [9], debate continues surrounding the lipid-lowering efficacy of low-fat vs moderate and high-fat PS-fortified products [10,11].

Considering the substantial increase in soymilk consumption in North America as an alternative to dairy milk, several studies have compared lipid-lowering responses of soymilk versus dairy milk [12-14]. While previous animal model investigations suggest that combination therapy of PS and soy protein reduces circulating cholesterol concentrations beyond that observed with either therapy alone [15], only one published report has investigated soymilk as a potential PS delivery beverage in human subjects; and this study was conducted without the advantage of a precisely controlled feeding design [16]. Furthermore, no information is available concerning the modulation of intestinal cholesterol absorption and synthesis in response to PS fortified soymilk. Given the reputed health benefits associated with the protein, isoflavone, saponin, and fiber components of soy [17], a unique opportunity exists to expand on these benefits through use of a PS fortified soy beverage. Here, we report on two separate controlled clinical feeding studies evaluating the efficacy of PS-enriched soy beverages differing in fat content on plasma lipids in comparison to a 1% dairy milk control. In study 1, our objective was to examine plasma lipids in response to the consumption of a low-fat (2 g/serving) PS-enriched soy beverage. In study 2, our objectives were to investigate plasma lipid and cholesterol kinetic responses following consumption of a moderate-fat (3.5 g/serving) PS enriched soy beverage.

## Results

### Study 1 - Low-Fat PS-Enriched Soy Beverage

Of the forty-two subjects initially recruited for study, 33 subjects (15 males and 18 females) completed both experimental phases. Nine subjects dropped out during the first phase due to difficulties with consuming study diets (n = 2), relocation to another city (n = 1), problems with daily centre visiting (n = 4), and personal reasons (n = 2). Palatability and texture of the PS-enriched soymilk beverage were assessed as highly acceptable. No side effects associated with soymilk or control milk consumption were reported.

Fasting endpoint lipid concentrations in response to low-fat PS-enriched soymilk consumption are presented in Table 1. TC concentrations were reduced (*P *< 0.0001) by 10% following consumption of the low-fat PS enriched soy beverage (203.40 ± 6.17 mg/dl; 5.26 ± 0.16 mmol/L) in comparison with the 1% dairy milk control (224.28 ± 6.57 mg/dl; 5.80 ± 0.17 mmol/L). Plasma LDL-C concentrations were reduced (*P *< 0.0001) by 13% following low-fat PS-enriched soymilk consumption (123.74 ± 5.80 mg/dl; 3.20 ± 0.15 mmol/L) in comparison with the 1% dairy milk (141.53 ± 5.80 mg/dl; 3.66 ± 0.15 mmol/L). Low-fat PS-enriched soymilk consumption decreased (*P *< 0.05) the LDL:HDL (2.74 vs 3.21) and TC:HDL (4.71 vs 5.05) ratios relative to 1% dairy milk. No treatment effects were observed in HDL-C and TG concentrations between the two groups.

**Table 1:** Fasting plasma cholesterol concentrations in hypercholesterolemic individuals in response to the consumption of a low-fat and moderate-fat plant sterol-enriched soymilk and 1% dairy milk for 29 days^a^

	***Study 1***		***Study 2***	
**Plasma lipids (mg/dl)**	**1% Dairy Milk**	**Low-fat Soymilk**	**1% Dairy Milk**	**Moderate-fat Soymilk**
Total cholesterol	224.28 ± 6.57	203.40 ± 6.19*	242.85 ± 10.44	216.16 ± 10.44^†^
LDL cholesterol	141.53 ± 5.80	123.74 ± 5.80*	162.41 ± 8.50	139.21 ± 7.73^†^
HDL cholesterol	47.17 ± 2.32	46.40 ± 2.32	47.56 ± 2.33	44.47 ± 2.71
LDL:HDL	3.21 ± 0.20	2.83 ± 0.18*	3.52 ± 0.25	3.18 ± 0.23^†^
Total:HDL	5.05 ± 0.26	4.71 ± 0.27*	5.38 ± 0.35	4.75 ± 0.33^†^
Triglycerides	178.03 ± 15.06	172.72 ± 16.83	185.12 ± 27.46	169.18 ± 25.69^†^

^a^Data presented as mean ± SEM, n = 33 for study 1; n = 23 for study 2.

*, significantly different from 1% dairy milk in study 1; ^†^, significantly different from 1% dairy milk in study 2.

Further analyses of TC and LDL-C were conducted in a subgroup (n = 14) of subjects with initial LDL-C concentrations above 3.4 mmol/L. Plasma TC concentrations decreased (*P *< 0.0001) by 9.5% following consumption of the low-fat PS-enriched soymilk, as compared to controls. Subjects consuming the low-fat PS-enriched soymilk had a 12% reduction in LDL-C concentrations (*P *= 0.0002), relative to the 1% dairy milk.

### Study 2 - Moderate-Fat PS Enriched Soy Beverage

Of the 32 subjects initially recruited for study, 23 completed (10 males and 13 females) both experimental phases. Of the 9 subjects who did not complete the trial, 3 dropped out during the initial days of the study citing an inability to comply with dietary restrictions. The 6 remaining dropouts left the study following completion of the first phase due to family (n = 2), health (n = 1), and work-related issues (n = 3). Palatability and texture of the PS-enriched soymilk beverage were assessed as highly acceptable. No side effects associated with soymilk or control milk consumption were reported.

Fasting endpoint lipid concentrations in response to PS-enriched moderate-fat soymilk consumption are presented in Table 1. TC was reduced by 12% (*P *< 0.0001) in the PS-enriched soymilk group (216.16 ± 10.44 mg/dl; 5.59 ± 0.27 mmol/L) in comparison with the 1% milk control (242.84 ± 10.44 mg/dl; 6.28 ± 0.27 mmol/L). Subjects receiving the PS-enriched soymilk displayed a 15% reduction (*P *< 0.0001) in LDL-C (139.21 ± 7.73 mg/dl; 3.60 ± 0.20 mmol/L) in comparison to 1% dairy milk (162.41 ± 8.50 mg/dl; 4.20 ± 0.22 mmol/L). PS-enriched soymilk consumption did not alter (*P *> 0.05) HDL-C concentrations. However, PS enriched soymilk consumption decreased (*P *< 0.05) endpoint LDL:HDL (3.18 vs. 3.52) and TC:HDL ratios (4.75 vs. 5.38) in comparison to the 1% dairy milk control. Subjects consuming the PS-enriched soymilk displayed a 9% reduction (*P *= 0.02) in plasma TG in comparison to those receiving the 1% dairy milk control.

**Figure 1:**
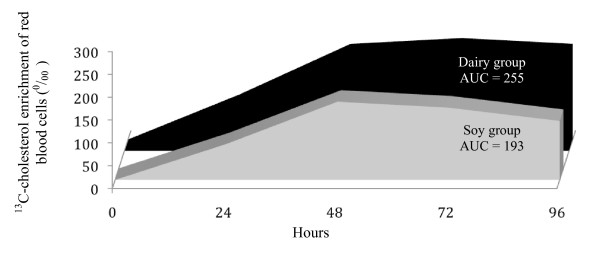
**^13^C-cholesterol enrichment of red blood cells at 24, 48, 72, and 96 h following ingestion of 75 mg of [3,4]-^13^C-cholesterol in response to consumption of moderate-fat plant sterol enriched soymilk**. Values are mean ± SEM (n = 17 for soy group, 18 for dairy group).

Cholesterol absorption, as measured by the area under the [3,4]-^13^C cholesterol RBC enrichment curve, was reduced (*P *= 0.02) by 27% in response to the consumption of PS-enriched soymilk in comparison to the 1% dairy milk control (Figure 1). In contrast, cholesterol synthesis, as measured by deuterium incorporation, did not differ (*P *= 0.45) between the PS-enhanced soymilk and 1% dairy milk groups (5.69 ± 0.63 vs 5.08 ± 0.79%/day).

## Discussion

Consumption of both low and moderate-fat PS enriched soy beverages reduced fasting total and LDL-C in comparison with 1% dairy milk. Although direct statistical comparison between the two clinical studies is not possible, the numerical plasma cholesterol responses between the two studies suggest that the fat content of the soymilk had little impact on vehicle performance as LDL-C reductions following consumption of the low and moderate-fat soymilk beverages were similar (13 and 15%, respectively). Direct comparisons of the clinical effectiveness of PS products between studies are often limited by differences in study design, as cholesterol reductions in response to PS consumption are modulated by multiple factors including background diet [18], PS dose [19] and form [20], frequency of PS consumption [21], level and type of fat in food vehicle [6], and baseline LDL-C concentration of study subjects [18]. As the background diet and factors pertaining to the dose, form and frequency of PS consumption were identical between our two studies, the comparable cholesterol-lowering response between the soymilk beverages may help clarify confusion that exists surrounding the effectiveness of PS enriched beverages with differing fat content. The only minor difference between our study protocols was the lower LDL-C baseline concentrations of the low-fat soy beverage group compared with the moderate-fat soymilk study subjects. It has been suggested that subjects with higher LDL-C may respond more favourably, and with further cholesterol reductions, than individuals with lower LDL-C concentrations [19]. However, when the analysis was conducted in hypercholesterolemic subjects with entry LDL-C concentrations above 3.4 mmol/L, TC and LDL-C reductions following PS-enriched low-fat soymilk consumption were similar to those reported for the entire study population. Therefore, our results suggest that patients with lower LDL-C concentrations may also benefit from the consumption of PS enriched soymilk.

Consumption of the moderate-fat PS enriched soymilk was associated with a 9% reduction in circulating triglycerides. Although PS consumption is not traditionally associated with modulation of triglyceride metabolism, Plat et al. recently observed a reduction in circulating TAG by 27.5% in metabolic syndrome patients with hypertriglyceridemia following the consumption of a PS yogurt drink [22]. Furthermore, this report follows a recent meta-analysis suggesting a PS-induced modulation of plasma triglycerides [23], although previous meta-analyses have failed to establish this relationship [6,19]. This PS-induced reduction in plasma triglycerides was only observed in subjects from study 2 who had elevated triglyceride concentrations compared to study 1 subjects, giving merit to Plat's speculation that modulation of triglycerides in response to PS consumption may be more readily detected in study populations with high baseline triglycerides.

**Figure 2:**
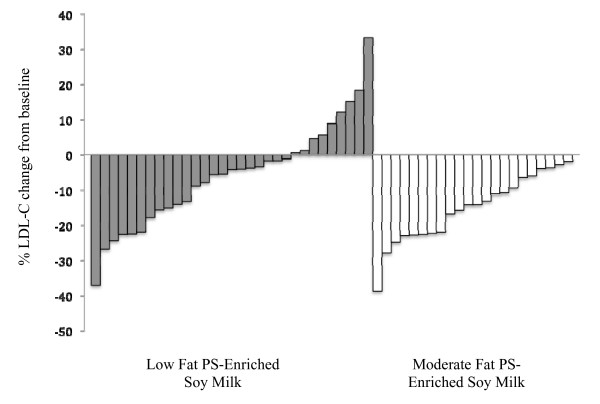
**Individual % changes in LDL-C from baseline in response to the consumption of a low and moderate-fat PS-enriched soy beverage**.

It is increasingly clear that some individuals respond with major shifts in lipid profiles to PS consumption whereas others are much less sensitive to PS challenges. This variability of responsiveness is likely associated with single nuclear polymorphisms (SNPs) in Niemann-Pick C1-like 1 (NPC1L1) and ATP binding cassette proteins G5 (ABCG5) and G8 (ABCG8), genes that regulate intestinal sterol absorption and efflux [24,25]. Although the two soymilk beverages were associated with similar LDL-C reductions, it is clear that the low-fat soymilk study population included more non-responders that subjects in the moderate-fat soymilk study (Figure 2). While consumption of the moderate-fat soy beverage was associated with an LDL-C lowering ranging from from 2 to 38%, LDL-C reductions following the low-fat soy beverage ranged from -33 to +37%, with 9 subjects displaying increased LDL-C following the low-fat soy treatment.

Cholesterol absorption was reduced by 27% following consumption of the moderate-fat PS fortified soy beverage. The cholesterol-lowering effects of PS are related to modulation of intestinal cholesterol absorption through several distinct mechanisms including interference with the micellular cholesterol incorporation [26], competition with dietary and biliary cholesterol for uptake through the brush border NPC1L1 [27], and reduction in cholesterol esterification and chylomicron assembly within the intestinal enterocyte [28]. Surprisingly, although previous investigations typically report compensatory increases in cholesterol synthesis in response to PS consumption [29,30], no difference was observed in cholesterol synthesis between the moderate-fat PS fortified soy beverage and control group in the current study. The hepatic expression of genes that regulate cholesterol synthesis and plasma LDL-C trafficking, including 3-hydroxy-3-methyl-glutaryl-CoA reductase, sterol-regulatory element binding protein 2, and LDL-receptor, have been shown to be transcriptionally regulated by individual protein and/or isoflavone components of soy [31,32]. Therefore, it can be speculated that specific bioactive components inherent in the soy beverage may have suppressed the increase in cholesterol synthesis typically observed following PS consumption. Alternatively, consumption of isolated soy protein has been reported to increase cholesterol FSR by 7.6% in hypercholesterolemic human subjects compared with animal protein consumption [33]. However, the effects of whole soymilk consumption on cholesterol synthesis has not been previously investigated.

## Conclusion

Dietary approaches effective in reducing LDL-C are critical to the development of sustainable health care systems. Incorporation of PS into a daily dietary regimen is an effective approach to improving dyslipidemia and potentially reducing the incidence of cardiovascular diseases. However, the food/beverage that is used to deliver an effective PS dose is an important factor affecting the cholesterol-lowering response. Results of this study suggest that, compared with 1% dairy milk, consumption of low and moderate-fat PS-enriched soymilk beverages are effective food based therapeutic strategies for cardiovascular disease management through reduction of plasma lipids and modulation of intestinal cholesterol absorption in normal to hypercholesterolemic subjects.

## Methods

### Subjects

Studies were conducted at the Clinical Nutrition Research Unit at the Richardson Centre for Functional Foods and Nutraceuticals (RCFFN), University of Manitoba. Subjects were recruited from the Winnipeg area using newspaper and radio endorsements, poster advertisements, and flyer distributions. Potential subjects, deemed eligible following an initial phone screening, visited the RCFFN for plasma cholesterol screening. Healthy males and females between the ages of 19-60 y with a body mass index (BMI) between 20-34 kg/m^2 ^were recruited who were free of thyroid disorders, kidney disease, diabetes mellitus, and heart disease, and were not currently taking lipid-lowering medications. Study 1 included normal to hypercholesterolemic subjects with a fasting plasma LDL-cholesterol (LDL-C) between 2.1-5.2 mmol/L and TG below 2.82 mmol/L. Study 2 included hypercholesterolemic subjects with a LDL-C greater than 3.4 mmol/L and TG below 2.82 mmol/L. Baseline characteristics of subjects who completed the study are presented in Table 2. To confirm individual health status, all subjects underwent a complete physical examination conducted by the study physician. The study protocols were approved by the Bioethical Research Ethics Board (BREB) at the University of Manitoba. All subjects signed informed consent to participate in the study.

**Table 2:** Baseline characteristics of subjects who completed the studies^a^

	***Study 1*^b^**	***Study 2*^c^**
Characteristic	Mean ± SE	Mean ± SE
*Anthropometric measurements*		
Age (years)	43.00 ± 2.37	43.9 ± 0.3
Height (m)	1.68 ± 1.83	1.66 ± 0.02
Initial body weight (kg)	83.21 ± 3.37	82.8 ± 4.0
Initial BMI (kg/m^2^)	29.08 ± 0.97	30.0 ± 1.5
*Plasma lipids (mg/dl)*		
Total cholesterol	210.36 ± 5.80	249.80 ± 11.60
LDL-cholesterol	129.54 ± 5.03	163.57 ± 8.12
HDL-cholesterol	47.56 ± 2.32	49.11 ± 2.70
Triglycerides	165.63 ± 15.05	206.37 ± 34.54

^a^Data presented as mean ± SEM; n = 33 for study 1; n = 23 for study 2

^b^Low-fat plant sterol-enriched soymilk study

^c^Moderate-fat plant sterol-enriched soymilk study

### Study design and protocol

Studies were carried out as 28-d controlled feeding trials using precisely prepared diets in a randomized crossover, two period design. Experimental periods were separated by a 4-wk washout period during which the subjects consumed his/her habitual diets. All meals were prepared at the Richardson Clinical Nutrition Research Unit as a 3-day rotating menu to provide the subjects with a variety of food during their experimental phases. Using a nutrient composition database (Food Processor, ESHA Research, Salem, OR), the diets were designed to contain 35% of energy as fat, 50% as carbohydrate and 15% as protein and meet the energy requirements of each subject using the Mifflin equation [34]. Subjects were randomly assigned to one of two experimental groups: the control group receiving 1% dairy milk or the treatment group receiving the fortified soy beverage providing 1.95 g PS/d (PS-enriched soymilks supplied by Whitewave Foods, Inc.) (Table 3). Enriched soy and 1% dairy milks were provided as 3 (240 ml) tetra-paks per day, 1 consumed with each meal. To monitor compliance, subjects consumed their daily supper meal in conjunction with one treatment or control beverage under direct supervision. The remaining meals and treatments were packed for take-out. Subjects were instructed to shake the tetra paks prior to drinking, consume only foods and beverages provided by the clinical research team, and return the empty containers to ensure that the beverage was properly administered.

**Table 3:** Nutrient composition of soymilk and 1% dairy milk^a^

		***Study 1***	***Study 2***
**Item**	**1% Dairy** **Milk Diet**	**Low-fat Soy** **Milk Diet**	**Moderate-fat Soy** **Milk Diet**
Calories (kcal)	100	80	100
Total fat (g)	2.5	2	3.5
Saturated fat (g)	1.5	0	0.5
Trans fat (g)	0	0	0
Total carbohydrate (g)	12	10	10
Fiber (g)	0	1	1
Protein (g)	8	6	6
Cholesterol	10	0	0
Plant sterols (g)	0	0.65	0.65

^a^Based on 1 serving size (240 ml)

### Outcome measurements

Twelve-hour fasting blood samples (26 ml) were collected on days 1 and 2 (baseline) and 29 and 30 (endpoint) during each phase. On day 24 of each experimental phase of study 2 (moderate-fat soymilk study only), a fasting baseline blood sample (0 h) was taken prior to administration of a 75 mg oral dose of [3,4]-^13^C cholesterol (CDN Isotopes, Point-Claire, Quebec, Canada) mixed in margarine and spread on an English muffin. Fasting blood samples were taken on day 25 (24 h), day 26 (48 h), day 27 (72 h), and day 28 (96 h) to measure cholesterol absorption. Additionally, following a fasting baseline sample on day 27, an oral dose of deuterium oxide (0.7 g/kg estimated body water) was given prior to breakfast as a tracer for measuring fractional cholesterol synthesis.

#### Plasma lipids

Plasma and red blood cells (RBC) fractions were separated by centrifugation at 3000 × *g *for 20 min and stored at -80°C. Plasma total cholesterol (TC), high-density lipoprotein cholesterol (HDL-C), and triglycerides (TG) were determined by automated enzymatic methods on a Vitros 350 chemistry analyzer (Ortho-Clinical Diagnostics, Markham, Ontario, Canada). LDL-C concentrations were estimated by the difference method using the Friedewald formula [35].

#### Cholesterol absorption

Cholesterol absorption was measured using the single stable isotope tracer approach with the area under the [3,4]-^13^C cholesterol RBC enrichment curve over 96 h representing cholesterol absorption. Free cholesterol was extracted from RBC according to established methods [36]. [3,4]-^13^C cholesterol enrichments in RBC lipid extracts were determined using on-line gas chromatography/combustion/isotope ratio mass spectrometry approach (Agilent 6890N chromatograph interfaced with a Finnigan Delta V Pulse isotope ratio mass spectrometer (Bremen, Germany). Isotope abundance, expressed in delta (d) per mil (‰), was calculated using CO_2 _as a reference gas and further corrected against the international reference standard, Pee Dee Belemnite limestone.

#### Cholesterol synthesis

Cholesterol fractional synthesis rate (FSR; %/day) was quantified using the uptake rate of deuterium from body water into the newly synthesized RBC free cholesterol pool over 24 h at the end of each study period [37]. Cholesterol FSR represents the fraction of the rapidly exchangeable central free cholesterol pool that exchanges between the plasma, liver, and intestine [38].

### Statistical analyses

The sample size of 20 subjects was calculated to detect an anticipated difference in LDL-cholesterol levels due to PS treatment of 12% (0.54 mmol/l) using an standard deviation of 0.732 mmol/l. The alpha and power were 0.05 and 0.7, respectively. All data are presented as means ± SEM. Statistical significance was set at *P *< 0.05 for all analyses. Differences in endpoint plasma variables were tested by repeated-measures ANOVA using baseline values as covariates for plasma lipid measurements. To examine the effect of baseline cholesterol concentrations on the lipid-lowering efficacy of PS-enriched soymilk consumption in study 1, further analyses of endpoint TC and LDL-C were conducted in a subgroup (n = 14) of subjects with initial LDL-C concentrations above 3.4 mmol/L. For cholesterol absorption, the area under the ^13^C-cholesterol enrichment curves from 24 to 96 h was calculated with the use of the trapezoidal rule after correction for baseline values. Data were analyzed with SPSS (version 16.0 for Mac, SPSS Inc, Chicago, IL).

## Competing interests

The authors declare that they have no competing interests.

## Authors' contributions

TCR contributed to the collection and analysis of data from study 2 and wrote the initial draft manuscript. YMC contributed to the collection and analysis of data from study 1 and contributed to the revision of the initial draft manuscript. SVH contributed to the analysis of the data and revision of initial draft manuscript. PJH contributed to the experimental design, served as a consultant on the clinical trial, and contributed to the revision of the initial draft manuscript.
